# The Relationship of Medical Assistants’ Work Engagement with Their Concerns of Having Made an Important Medical Error: A Cross-Sectional Study

**DOI:** 10.3390/ijerph19116690

**Published:** 2022-05-30

**Authors:** Adrian Loerbroks, Patricia Vu-Eickmann, Annegret Dreher, Viola Mambrey, Jessica Scharf, Peter Angerer

**Affiliations:** Institute of Occupational, Social, and Environmental Medicine, Centre for Health and Society, Faculty of Medicine, University of Düsseldorf, Universitätsstr. 1, 40225 Düsseldorf, Germany; patricia.vu-eickmann@uni-duesseldorf.de (P.V.-E.); annegret.dreher@med.uni-duesseldorf.de (A.D.); viola.mambrey@uni-duesseldorf.de (V.M.); scharfje@uni-duesseldorf.de (J.S.); peter.angerer@uni-duesseldorf.de (P.A.)

**Keywords:** health care staff, Germany, medical assistants, medical errors, work engagement

## Abstract

Objectives: We aimed to examine associations of work engagement with self-reported concerns of having made medical errors among medical assistants. Methods: We used cross-sectional questionnaire data from 424 medical assistants in Germany (collected between March and May 2021). The nine-item Utrecht Work Engagement Scale assessed the subdimensions vigor, dedication, and absorption. Participants further reported whether they were concerned that they had made an important medical error in the last three months. Work engagement scores were used both as categorized variables (i.e., highest tertile vs. remaining tertiles) and continuous variables (i.e., z-scores) and their associations with concerns to have made an important medical error were examined using multivariable logistic regression to estimate odds ratios (ORs) and corresponding 95% confidence intervals (CIs). Results: High vigor (versus low vigor) and high dedication (versus low dedication) were associated with substantially reduced odds of expressing concerns to have made an important medical error (OR = 0.19, 95%CI = 0.04–0.85 and OR = 0.25, 95%CI = 0.07–0.88, respectively), but absorption was not (OR = 1.10, 95%CI = 0.43–2.86). Analyses with z-scores confirmed this pattern of associations for vigor and absorption, but less so for dedication (OR = 0.72, 95%CI = 0.47–1.11). Conclusions: Vigor and possibly also dedication are inversely related to concerns of having made an important medical error. Our findings may suggest that promotion of these subdimensions of work engagement may improve patient safety.

## 1. Introduction

Working conditions in health care have been characterized as unfavorable, for instance, in terms of time pressure, restricted rewards, the need for multitasking, low job control, and interpersonal conflicts [1,2]. Those adverse working conditions have been identified as risk factors for poor mental health [3,4], and accordingly, mental health problems are common among health care staff, including the burnout syndrome [1,5]. Burnout has been found to compromise functioning at work in health care staff [6,7], and accordingly, relationships of burnout with poorer patient care and patient safety have been repeatedly documented [8,9,10,11]. An implication of those findings is that patient care and safety could be improved through the prevention of burnout (i.e., a pathogenic approach).

An alternative to the above-mentioned pathogenic approach would be to improve the quality of patient care through the promotion of workers’ psychosocial resources (i.e., a salutogenic approach). One such resource—which may be viewed as the beneficial counterpart of burnout [12]—is work engagement. Work engagement is considered to comprise three dimensions referred to as vigor (i.e., high energy levels and work-related persistence), dedication (i.e., pronounced emotional involvement in and enthusiasm about one’s work), and absorption (i.e., being fully focused on and completely immersed into one’s job with the perception that time flies) [12]. Recent evidence suggests that work engagement predicts better mental health [13] as well as reduced sickness absence [14] and that it is inversely associated with turnover intentions in health care staff [15]. It has further been found that work engagement promotes occupational functioning and performance [16,17]. Accordingly, work engagement has been associated with better quality of care [18]. A key dimension of the quality of care is patient safety [19]. Patient safety refers to the minimization of harm to patients and a key indicator of patient safety are medical errors [19]. A recent meta-analysis showed that work engagement is inversely related to errors in health care staff [20]. A limitation of the available evidence is though that prior studies on this topic were exclusively conducted in hospital settings and only among two professional groups (i.e., nurses and physicians). One may assume, for instance, that the types of tasks and thus the nature of potential errors, their frequency, and their potential impact on patient safety vary widely among different types of health care professions (e.g., physicians, nurses, or allied health professionals) and across settings (e.g., hospitals vs. primacy care). In addition, work engagement may differ between health care professions: work engagement is considered to be primarily shaped by job resources [12,21] and such resources seem to vary across types of health care professions (e.g., in terms of job control, social support, promotion prospects, financial reward, or prestige) [22,23,24]. Consequently, research in additional health care settings and a wider range of occupational groups has been called for [20].

To respond to this call, we aimed to examine potential associations of work engagement with potential medical errors specifically among medical assistants. In Germany, medical assistants represent the largest professional group in primary care. Upon completion of three years of vocational training, medical assistants in German health care are mainly involved in the management of medical practices and patients in order to support the work of physicians. Medical assistants are an almost entirely female professional population [25]. Their duties are both clinical and administrative. Administrative tasks may include, amongst others, the scheduling of appointments, documentation, and management of official correspondence and of consumables. Medical assistants’ clinical tasks may cover a broad range and largely involve performance of standardized diagnostic procedures as delegated by the supervising physician. Examples include the performance of laboratory tests, spirometry, X-rays, or electrocardiography, administration of injections, wound care, drawing blood samples, and taking standardized patient histories [26]. Errors related to those tasks can result in harm to patients. For instance, the incorrect application of diagnostic procedures may lead to false-negative test results and thus failure to initiate the required treatment. In addition, inadequate administration of therapeutic measures by medical assistants may put patients at an increased risk of complications (e.g., incorrect administration of injections in terms of unhygienic procedures). Errors related to diagnosis and prescriptions have been identified as the incidents that are most likely to result in severe harm of patients in primary care [27]. However, medical assistants’ administrative duties may also have unfavorable implications for patients’ health: medical assistants are the first contact person for patients in practices and are expected to adequately assess the severity of patients’ complaints and to prioritize such complaints when scheduling access to the physician. Misjudging the urgency of the patients’ complaints when they contact the practice has been identified as one of the most severe potential errors in general practitioner practices [28].

To sum up, the available evidence on the link between work engagement and medical errors builds on studies among physicians and nurses in hospitals, and therefore, the generalizability across health professions and health care settings is limited [20]. The objective of this study was thus to provide, for the first time, evidence for medical assistants who are a professional group usually working in primary care in Germany.

## 2. Methods

### 2.1. Study Population

We drew on cross-sectional data from the follow-up assessment of a cohort study of medical assistants in Germany. Briefly, between September 2016 and April 2017, we carried out the baseline survey [2,29,30]. We collected data mainly through an online survey; however, medical assistants could also request to receive hard copies for completion. Recruitment of participants was carried out nationwide in Germany and with the support of various multipliers and numerous types of communication channels. Amongst others, we included fliers in the members magazine of the Association of Medical Professions (VMF e.V.), which represents medical assistants. We further advertised our study at various regional Associations of Statutory Health Insurance Physicians via internal distribution, home pages, or direct forwarding to medical practices or medical assistant schools. Recruitment of medical assistants was also carried out by presenting our study at relevant conferences and training events. In total, 944 MAs completed the baseline questionnaire.

The follow-up data collection was carried out between March and May 2021. Participants were again invited to complete an online survey or hard copies of the questionnaire. In total, 537 MAs (56.9%) participated at the follow-up. For the current analysis, we used cross-sectional data from the follow-up assessment and included only participants who reported to be in current employment as a MA at follow-up (n = 424) (e.g., in contrast to current employment but not as a MA, unemployment, parental leave, or retirement). We decided to use the follow-up data for cross-sectional analyses for two reasons: First and foremost, the psychosocial working conditions and possibly also the work engagement of MAs have dramatically changed between baseline assessments (i.e., in 2016/2017) and follow-up (i.e., 2021) due to the COVID-19 pandemic [31]. As a consequence, any findings based on our cross-sectional baseline data may not be generalizable to the COVID-19 era. Further, prospective analyses may have limited validity, as the perception of psychosocial working conditions and work engagement cannot be assumed to have been stable throughout the follow-up period. A second reason to use follow-up data is that we measured only two dimensions of work engagement at baseline (i.e., absorption was omitted), but all three dimensions at follow-up. Written informed consent was obtained from all participants. Our study was approved by the Ethics Committee of the Medical Faculty of the Heinrich-Heine-University of Düsseldorf (ethic registration number: 2019-819).

### 2.2. Measures

We measured work engagement in terms of its subdimensions vigor, dedication, and absorption using the German version of the 9-item Utrecht Work Engagement Scale (UWES) [32]. Either subscale consists of three items. Participants rated the perceived frequency of the described experiences using a 7-point Likert scale varying between “never” (=0) and “always/every day” (=6). We calculated mean scores for vigor, dedication, and absorption whereby higher scores represent higher levels of the respective construct. The internal consistency was good as suggested by Cronbach’s alphas of 0.86 and above for each subscale.

We assessed concern of having made an important medical error by the following single item: “Are you concerned that you have made an important medical error in the last three months?”. Response options were yes or no. This item has been used in prior research [33].

Socio-demographic and occupational data used for the current study included self-reported age, sex (female/male/divers), occupational standing (reflected by participants’ reports to hold a leadership position versus reporting not to hold such a position), and working experience. We measured the latter by an item inquiring after the number of years that the respondent has worked as a medical assistant since graduation.

### 2.3. Statistical Analyses

The exposure variables (i.e., vigor, dedication, and absorption) were used both as categorized variables (i.e., highest tertile vs. remaining tertiles) and continuous variables (i.e., z-scores). Those variables’ associations with expressed concerns of having made an important medical error were examined using separate logistic regression models to estimate odds ratios (ORs) and corresponding 95% confidence intervals (CIs). We first ran unadjusted models and then corrected our estimations for age and sex, and subsequently in addition for holding a leadership position and working experience.

## 3. Results

As shown in Table 1, the mean age was 46.77 years, but participant age displayed wide variation (standard deviation [SD] = 10.39). With few exceptions, participants reported to be female and about every second participant reported to hold a leadership position. The mean working experience as a medical assistant equaled 23.94 years and showed a wide spread (SD = 11.34). Given their potential score ranges, vigor, dedication, and absorption were at intermediate levels. In total, 23 (5.40%) medical assistants reported to be concerned that they had made an important medical error during the prior three months.

Logistic regression analyses (see Table 2) suggested that both high vigor (versus low vigor) and high dedication (versus low dedication) were associated with substantially reduced odds of reporting concerns about having made an important medical error (OR = 0.19, 95%CI = 0.04–0.85 and OR = 0.25, 95%CI = 0.07–0.88, respectively). In contrast, absorption was not associated with the outcome (OR = 1.10, 95%CI = 0.43–2.86). Analyses with z-scores confirmed this pattern of associations for vigor and absorption, but less so for dedication (OR = 0.72, 95%CI = 0.47–1.11).

## 4. Discussion

Our study of medical assistants in Germany suggests that vigor and potentially dedication, but not absorption, show inverse associations with concerns of having made an important medical error. Evidence for the association between work engagement and medical errors remains sparse and is limited to physicians [34,35,36] or nurses [37,38] in hospital settings. All those prior studies consistently suggested associations of work engagement across a wide range of errors, such as medication errors, documentation errors, nosocomial infections, and suboptimal practices (e.g., delayed patient discharge due to a high workload) [35,37,38]. While three prior studies used the UWES to measure work engagement [34,35,36], only two examined subdimensions of work engagement [34,35]. Both of those studies suggested that in particular the subcomponents vigor and dedication—rather than absorption—are associated with suboptimal patient care [34,35]. Thus, overall, our findings confirm the inverse relationships between work engagement (i.e., vigor and dedication) and medical errors for the first time for the professional group of medical assistants who represent the largest professional group in primary care in Germany.

Medical assistants who experience high work engagement may make fewer errors due to their more committed pursuit of learning opportunities [12] and thus possibly better medical knowledge [39] or more up-to-date knowledge and skill sets. Additionally, one may speculate that engaged health care staff is able to deliver patient care with fewer errors because of the willingness and capability to adhere more closely to safety protocols. Assuming that work engagement represents the beneficial counterpart of burnout [12], one may hypothesize based on the burnout literature that medical assistants with high work engagement are more committed and/or better able to contribute to effective teamwork [40], which has been found to predict patient safety [40]. Furthermore, there may be broader conditions related to medical assistants’ professional settings that may cause both increased work engagement and better quality of care, such as leadership styles [41,42]. Errors may be corrected instantly by engaged medical assistants, which implies that concerns about important medical errors are reported less frequently in a survey. We cannot rule out though that work engagement is related to reduced odds to report errors, because medical assistants with high work engagement and thus a very positive experience of their job-related duties are less inclined to notice or report medical errors [35].

A limitation of our study is its cross-sectional design. Such studies do not provide insights into the potential temporal sequence of associations, which is considered a key criterion to establish causality [43]. Another limitation of our study is that we were unable to estimate the response rate. However, it needs mentioning that the characteristics of our study population are comparable to those in a representative study of medical assistants in Germany [44], except for age (i.e., our participants were older, that is, 46.8 years versus 37.4 years). We cannot rule out though that participation was related to other factors that are relevant to the current study (i.e., high work engagement), which would limit the generalizability of our findings. Another point to consider is the fairly small sample size of our study and the low number of outcomes (n = 23). The former implies limited statistical power and the latter implies that only a few confounders can be considered to make valid statistical estimations. It is reassuring in this respect that unadjusted models showed similar patterns of associations compared to multivariable-adjusted models. However, larger studies are needed to provide more precise estimates.

A final possible limitation is our approach to measure errors in terms of *concerns* of having made important medical errors by self-reports, which has been used in prior research [33]. Firstly, the inquiry after concerns of having made medical errors rather than certainty of having made an error is associated with some strengths and weaknesses. By referring specifically to concerns rather than noticed actual errors, social desirability and fear of potential legal consequences may be reduced, and thus likewise, the threshold to report any error is increased. The fear of legal action, which is a reason not to disclose medical errors [45], may be further diminished by the fact that we communicated to participants in our recruitment materials that data are treated confidentially and in line with all relevant legal regulations. The disadvantage of inquiring after concerns is that reports may include medical errors that have never actually been made. Second, participants were not provided with a definition of what constitutes an important medical error. We can thus not assume a common understanding of this concept. This may have resulted in misclassification, which was likely non-differential and therefore would have diluted the magnitude of the true associations between work engagement and concerns of having made an important medical error. Finally, we assessed concerns of having made errors as a potential proxy of errors based on self-reports, which is common [8]. An assessment of errors based on sources other than self-report (e.g., through observations, screening of records, or follow-up surveys of patients to inquire after complications) was beyond the resources of our study. Objective assessments had been desirable though as a complementary measure, because it has been suggested that wellbeing constructs in health care professionals are often associated with self-reported quality of care indicators, but not with objective markers [9]. However, this discrepancy does not necessarily imply that sources that are considered more objective are superior or provide estimations of errors that are free of bias [46,47]. Further, the assessment of (concerns of) errors based on self-report offers certain advantages, i.e., firstly, such measures are not confined to specific types of errors [8], and second, the collection of such data is associated with lower cost, which is relevant in large-scale studies. In fact, it has been suggested that the lack of association between wellbeing constructs such as burnout and objective markers of the quality of care or errors in some studies may partly be explained by the fact that those studies were too small and thus statistically underpowered [9]. While the inquiry after medical errors in general represents a pragmatic methodological approach, it had been of interest to collect additional data on the types of errors. Insights into specific types of errors and their possible prevalence would be particularly useful to devise preventive strategies aiming to increase patient safety.

In our study, only 5.4% of the participants reported concerns about having made an important medical error in the last three months. Yet, such errors can have severe consequences for patients’ health (e.g., misdiagnoses due to inadequately performed procedures). If our findings are corroborated by future prospective research and the suspected direction of causality is confirmed (i.e., work engagement affecting [concerns about having made] medical errors), interventions could be devised to improve patient safety through the promotion of work engagement. This salutogenic and health-promoting approach could supplement a pathogenic approach that seeks to improve patient safety through prevention of burnout [8,9,10,11]. Work engagement is supposed to be mainly shaped by job resources [12]. An important job-related resource as perceived by medical assistants is their close, empathic, rewarding, and (long-standing) social contact with patients [48]. Additional perceived key resources are social support at work (e.g., from colleagues) in light of high job demands, the diversity of tasks (e.g., medical, social, administrative), and high job control related to their own set of defined key tasks (e.g., sole responsibility for the laboratory) [48]. Those resources could be enhanced to improve work engagement. Job control and one’s responsibility for a distinct set of tasks could be promoted, for instance, through novel approaches to delegate tasks from physicians to medical assistants. In Germany, medical assistants can complete courses to work as a so-called care assistant in physicians’ practices (VERAH^®^). Such VERAHs are, for instance, permitted to pay home visits to patients without their supervising physician.

## 5. Conclusions

To sum up, we observed that work engagement in terms of vigor and dedication shows inverse associations with concerns of having made an important medical error among medical assistants in Germany. If confirmed by prospective evidence, these findings may suggest that the promotion of work engagement may be conducive to patient safety.

## Figures and Tables

**Table 1:** Characteristics of the study population (n = 424).

Characteristics	
Age, mean (SD ^†^)	46.77 (10.39)
Female (vs. non-female), n (%)	418 (98.6)
Holding a leadership position, n (%)	206 (48.60)
Working experience as a medical assistant (years), mean (SD ^†^)	23.94 (11.34)
Vigor (potential score range = 0–6), mean (SD ^†^)	3.17 (1.34)
Dedication (potential score range = 0–6), mean (SD ^†^)	3.54 (1.37)
Absorption (potential score range = 0–6), mean (SD ^†^)	3.50 (1.47)
Concerns about having made an important medical error in the last three months, n (%)	23 (5.40)

^†^—SD = standard deviation.

**Table 2:** The association of the work engagement subdimensions vigor, dedication, and absorption with concerns about having made an important medical error in the last three months (n = 424).

		Unadjusted		Age-And-Sex-Adjusted		Multivariable Adjusted ^†^	
		OR ^‡^	95% CI ^§^	OR ^‡^	95% CI ^§^	OR ^‡^	95% CI ^§^
Vigor	Low	1.0	reference	1.0	reference	1.0	reference
	High	0.19	0.04, 0.81	0.19	0.04, 0.83	0.19	0.04, 0.85
	z-score	0.52	0.32, 0.83	0.51	0.32, 0.83	0.52	0.32, 0.85
Dedication	Low	1.0	reference	1.0	reference	1.0	reference
	High	0.26	0.08, 0.89	0.25	0.07, 0.88	0.25	0.07, 0.88
	z-score	0.71	0.46, 1.08	0.70	0.46, 1.08	0.72	0.47, 1.11
Absorption	Low	1.0	reference	1.0	reference	1.0	reference
	High	0.98	0.39, 2.44	0.96	0.38, 2.41	1.10	0.43, 2.86
	z-score	0.81	0.53, 1.23	0.80	0.52, 1.22	0.83	0.54, 1.28

^†^—Adjusted for age, sex, leadership position, and years of working experience; ^‡^—OR = Odds ratio; ^§^—CI = Confidence Interval Cut-off = highest tertile vs. remaining tertile.

## Data Availability

The data are available from the corresponding author upon reasonable request.
